# High Resolution Quantitative Angle-Scanning Widefield Surface Plasmon Microscopy

**DOI:** 10.1038/srep20195

**Published:** 2016-02-01

**Authors:** Han-Min Tan, Suejit Pechprasarn, Jing Zhang, Mark C. Pitter, Michael G. Somekh

**Affiliations:** 1 Intellectual Property Office Ministry of Economic Affairs, Da-an, Taipei, Taiwan; 2 Department of Electronic and Information Engineering, the Hong Kong Polytechnic University, Hung Hom, Kowloon, Hong Kong SAR; 3 Keele University, Guy Hilton Research Centre, Thornburrow Drive, Hartshill, Stoke-on-Trent, Staffordshire, ST4 7QB UK; 4 Natural Power, The Old Barns, Fair Oaks Farm, Hollybush, HR8 1EU, UK

## Abstract

We describe the construction of a prismless widefield surface plasmon microscope; this has been applied to imaging of the interactions of protein and antibodies in aqueous media. The illumination angle of spatially incoherent diffuse laser illumination was controlled with an amplitude spatial light modulator placed in a conjugate back focal plane to allow dynamic control of the illumination angle. Quantitative surface plasmon microscopy images with high spatial resolution were acquired by post-processing a series of images obtained as a function of illumination angle. Experimental results are presented showing spatially and temporally resolved binding of a protein to a ligand. We also show theoretical results calculated by vector diffraction theory that accurately predict the response of the microscope on a spatially varying sample thus allowing proper quantification and interpretation of the experimental results.

Surface plasmon resonance (SPR) occurs in thin metallic films on dielectric substrates when the *k*-vector of the incident light matches that of *k-*vector of surface plasmons (SPs). SPR is characterized by a sharp decrease in reflectivity at the resonant angle and the value of this angle is very sensitive to the conditions immediately adjacent to the metal film, in the present case the metal used is always gold, since it is inert and gives a strong SP response. This has allowed SPR to be widely applied to monitor thin film formation^1^ and the binding of target molecules to ligands on the gold surface^2^.

Most current applications of SPR are non-imaging, but spatially resolved SP microscopy^3^ was first reported in 1987 using a prism based Kretschmann configuration. In this arrangement, the spatial resolution of reflection mode (brightfield) images using a CCD is limited due to the long working distance and the resultant low numerical aperture (NA) of the objective lens. The prism also distorts the SPR images and the oblique viewing angle can cause the images to move as the incident angle of the illumination is varied.

The use of a high NA microscope objective to excite SPs has been reported by Kano^4^. The configuration used is similar to total internal reflection fluorescence microscopy^5^ and gives better lateral resolution compared to prism based systems. Their implementation was a point-scanning system and the image was obtained by processing the Fourier spectrum in the back focal plane (BFP) of the microscope objective. Other point scanning techniques based on modified confocal microscopy^6^,^7^,^8^ or tracking the back focal plane have been developed recently and show great promise to obtain extremely high sensitivity^9^,^10^,^11^, however, they are relatively slow compared to the widefield approach used in the present paper when multiple detection points are measured.

Examples of widefield microscopes adapted for SP measurement have been presented by Stabler *et al.*^12^ and Zhang *et al.*^13^ which used a Köhler illuminated high-resolution widefield microscope to obtain widefield SPR images and while the images showed good contrast they were not quantitative since the excitation angle of the SPs was not recovered. This microscope system has, however, been used to obtain results of antibody/antigen binding by comparing the contrast between different regions on the sample. This method was, however, only capable of measuring end-point binding since the sample was dried at each measurement point. Although this work was laborious and not practical for routine use it did demonstrate the potential of the widefield SPR microscope for binding studies^2^.

The most similar approach to the one presented in this paper was described by Huang *et al.*^14^ where they focused a laser beam onto the back focal plane of the objective lens and moved the illumination point mechanically to change the angle of illumination onto the sample. In the present paper we use a spatial light modulator (SLM) in the back focal plane of a microscope to control the illumination.

The present paper makes the following four significant improvements and innovations over previous publications:

- We discuss how the SLM illumination gives greatly improved imaging performance compared to illumination at a single point in the back focal plane^14^. The use of a spatially incoherent source leads to imaging considerably more free of artefacts, with far better spatial localization. This is demonstrated by modelling with rigorous coupled wave diffraction analysis (RCWA)^9^,^10^,
- we show how the local structure affects the measurement of refractive index, and we apply a full diffraction model rather than using the naïve approach of simply considering the Fresnel equations appropriate for a uniform substrate. This allows us to compare the response in both experiment and theory in order to make truly quantitative measurements,
- we present results showing protein binding in aqueous media, which has not been presented in previous work^9^,^10^ and
- we quantify refractive index sensitivity which while less good that single point measurements is good for a widefield configuration^15^.

We believe that the ability to make multiple quantitative measurements with a relatively simple modification to a conventional widefield microscope offers exciting measurement potential that is normally only met with far more specialist instrumentation.

## Results

In this section we present results that demonstrate points (1) to (4) listed at the end of the previous section.

The microscope system used for the experimental results and modelled in this paper is essentially a modified widefield microscope with Kohler illumination. The SLM is conjugate to the back focal plane and by projecting an annular ring the range of illumination angles onto the sample is controlled. The oil immersion objective provides sufficient range of illumination angles to excite SPs on a gold substrate. Varying the illumination with the SLM allows these angles to be changed so that the angle at which the SPs are excited at each position can be observed at each spatial position. The details of the optical system and the processing procedure are discussed in the Methods section.

### Simulations to compare point excitation with ring excitation

**Figure 1:**
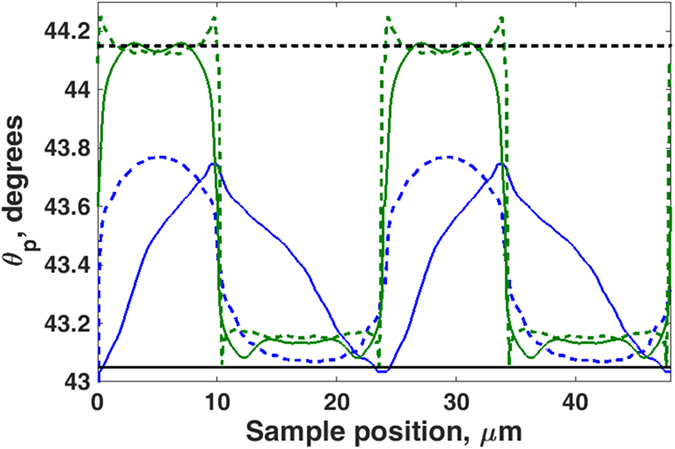
Shows simulated plasmonic angles recovered from full annular illumination and point scanning illumination described in Huang *et al.*^14^. The incident wavelength was 690 nm and the sample was a 24 micron period grating deposited on 50 nm of gold with 2 nm of Cr adhesion layer. The black line and the dotted black line represent bare gold case and uniform layer of 14 nm thick of BSA coated on uniform gold respectively. The blue and dotted blue lines represent the point illumination and annular BFP illumination where the sample was a grating deposited on gold with the grating vector direction parallel to the SP propagation direction. The green and dotted green lines represent the point illumination and annular BFP illumination where the sample was a grating deposited on gold with the grating vector direction parallel to the SP propagation direction.

We use RCWA simulations (i) to demonstrate the advantages of ring illumination and (ii) to interpret and process the experimental results discussed later in this section. The details of the RCWA simulations are described in more detail in the Methods section. In this subsection we simulate the response using point illumination in the back focal plane to compare with ring illumination in the same plane as shown in Figure 1. In each case we follow the protocol described in the Methods section; that is the incident angle was scanned and the minimum angle recovered was assigned to the pixel value at that location. The sample used in the simulations matched those used in subsequent experiments. The sample consisted of a plane gold layer (50 nm thick) with a thin 2 nm adhesion of chromium on a glass substrate. A bovine serum albumin (BSA) grating of period 24microns and thickness 14 nm was deposited on the gold layer. The results at 690 nm wavelength are shown in Figure 1 for the SP propagating parallel to the grating (perpendicular to the grating vector), we can immediately see that both point illumination and ring illumination recover values close to those recovered on a uniform surface, however, the transition is sharper with the ring, essentially because of the wider range of incident spatial frequencies, for shorter grating periods the benefits of this sharper transition become more apparent. When the SPs propagate across the grating we see that the point illumination gives an asymmetrical image which displaces the image by moving the center of gravity for each region. Moreover, the transition between regions is less steep making it much more difficult to recover the local values of index.

### System calibration

**Figure 2:**
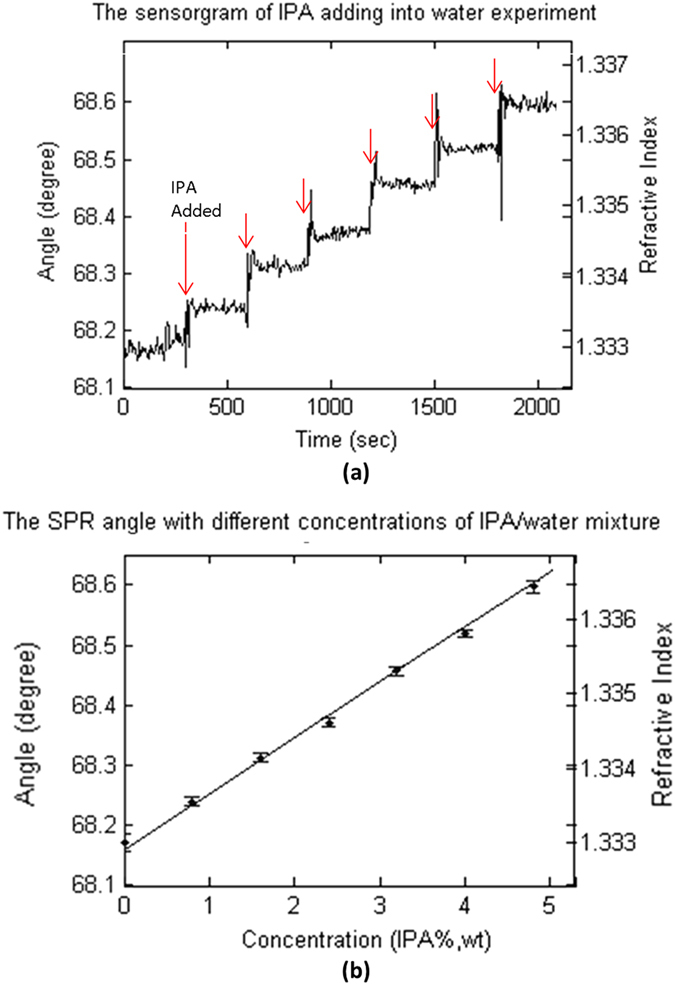
On-line monitoring of the SPR angle as a function of IPA/water mixture concentration (**a**). The SPR angle against time when changing the mixture concentration from 0% (wt) to 4.8% (wt.). The concentration difference of each step is 0.8% (wt.) (**b**) The SPR angle vs. the concentration of IPA/water mixture (% IPA, wt).

The first experiment whose results are shown in Fig. 2 was performed for two principal purposes (i) to quantify the system performance and (ii) to obtain best fit parameters for the metal layer to use in subsequent simulations and data recovery. The experiments were performed on a uniform gold surface whose fabrication is discussed in the Methods section (sample a).

Figure 2 demonstrates the sensitivity and quantitative nature of the angle-scanning SPR microscope by analyzing sample (a). The SPR angle of Fig. 2a is the mean value obtained from a region of 625 pixels (4.7 × 4.7 μm^2^) of the ASWSPRM (Angle Scanning Widefield Surface Plasmon Resonance Microscope). The standard deviation of the measurements, at each concentration was determined, and after removing constant variation due to drift the root-mean-square (RMS) noise was determined to be 3.97 × 10^−3^ deg. This corresponds to a refractive index change of 3.4 × 10^−5^ RIU.

From simulation the parameters of the metal film that best fit these results is Cr (index 3.6250+4.2596i^16^, thickness 2 nm), Au (index 0.1985+4.5400i, thickness 50 nm). The value of the imaginary part of the refractive index of gold is slightly greater than the values in the literature (0.1985+4.1400i^17^), we believe these differences can readily be accounted for by surface finish and grain structure of the layers.

### Images on grating samples and binding experiments

**Figure 3:**
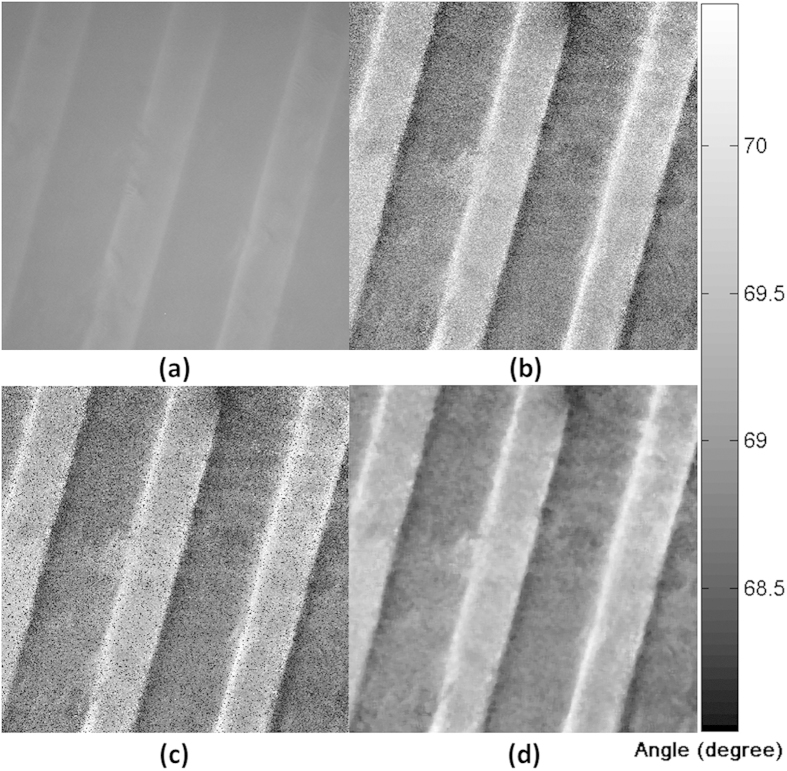
Images of microcontact printed BSA grating sample in water with different processing methods. The field of view is 70 μm × 70 μm, the period of grating is 24 μm. (**a**) Image taken at a fixed incident angle of 68 degrees. (**b**) Image obtained by selecting the minimum value (**c**) Using 9 point fitting a 3^rd^ order polynonial at each pixel and calculate the resonant angle by differentiating and calculating the minima. (**d**) As Fig.3(c) after applying a 5 × 5 median filter.

Figure 3 shows the effect of different processing methods to quantify ASWSPRM response on the microcontact printed samples (samples b described in the Methods section) described above when exposed in water. Figure 3a is obtained for a fixed incident angle of 68 degrees, it shows high resolution but low contrast and is not quantitative. Figure 3b (simply choosing minima value) gives a less precise numerical value compared to Figure 3c,d where a cubic curve fit is performed, this simple approach can, however, be used to get initial results during experiments. In Figure 3c, there are some black spots on the image, which are the outliers due to noise. Figure 3d was obtained using a cubic curve fit and a 5×5 pixel median filter to remove outliers, which gives good quantification over the whole image.

**Figure 4:**
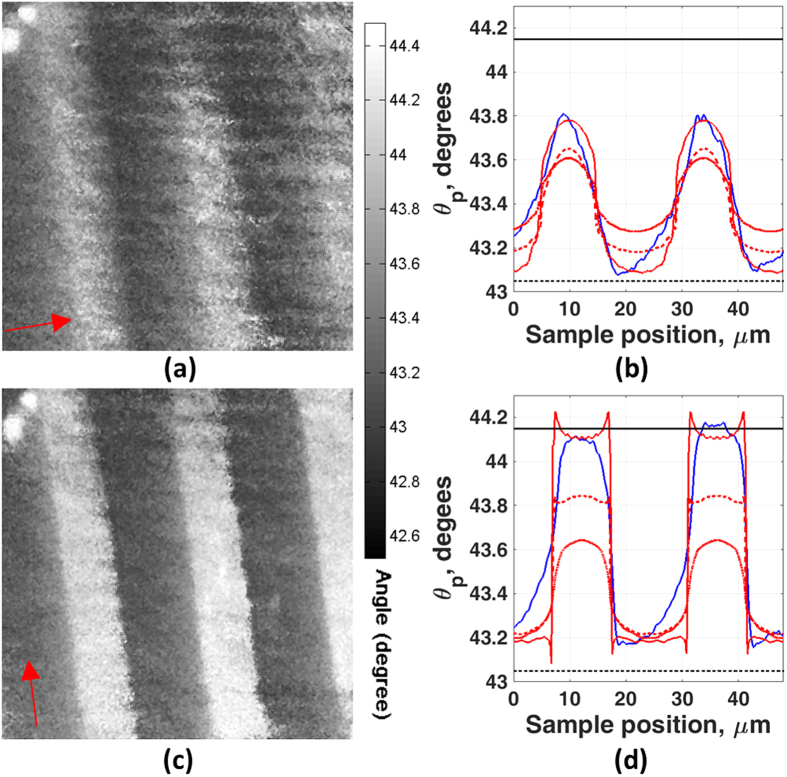
Images of BSA grating sample with different incident light polarization in air. The field of view is 70 μm × 70 μm, the period of grating is 24 μm. (**a**) Grating vector parallel to incident polarization direction (arrow indicates nominal polarization direction). (**b**) Linescan image of case (**a**) compared with simulation result. (**c**) Grating vector perpendicular to incident polarization direction (arrow indicates nominal polarization direction). (**d**) Linescan image of case (**c**) compared with simulation result. The black line and the dotted black line represent uniform layer of 14 nm thick of BSA coated on uniform gold and bare gold case respectively. The blue curve shows experimental linescan image. The dotted-dash red, dotted red and red curves represent the simulation results where the sample was BSA grating with 10 nm, 12 nm and 14 nm thick respectively.

**Figure 5:**
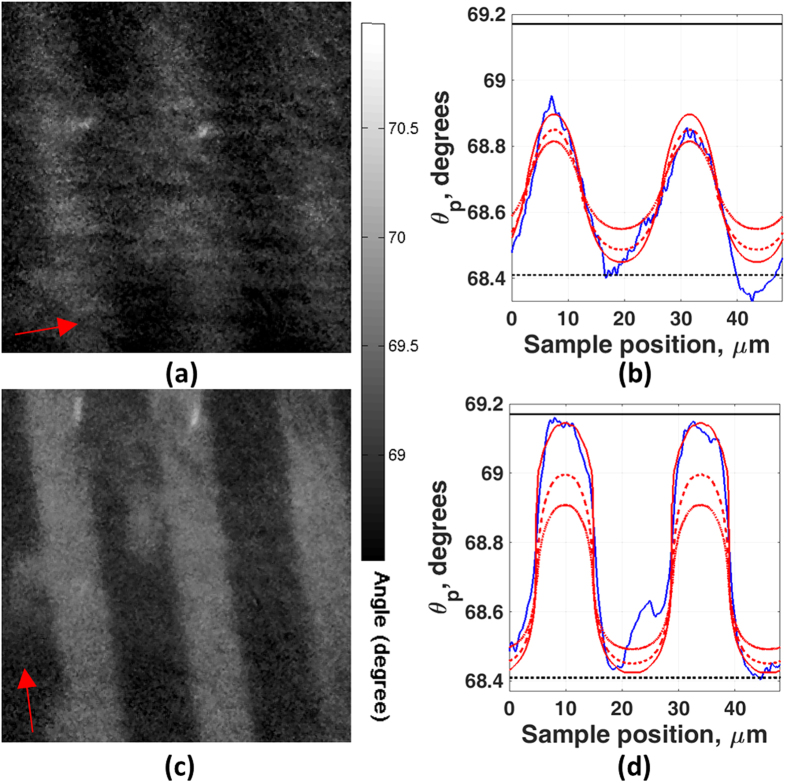
Images of BSA grating sample with different incident light polarization. The field of view is 70 μm × 70 μm, the period of grating is 24 μm. (**a**) Grating vector parallel to incident polarization direction and the sample exposed in the water (arrow indicates nominal polarization direction). (**b**) Linescan image of case (**a**) compared with simulation result. (**c**) Grating vector perpendicular to incident polarization direction and the sample exposed in the water (arrow indicates nominal polarization direction). (**d**) Linescan image of case (**c**) compared with simulation result. The black line and the dotted black line represent uniform layer of 14 nm thick of BSA coated on uniform gold and bare gold case respectively. The blue curve shows experimental linescan image. The dotted-dash red, dotted red and red curves represent the simulation results where the sample was BSA grating with 10 nm, 12 nm and 14 nm thick respectively.

Figures 4 and 5 show results from a plane gold sample on which a protein grating sample produced by microcontact printing was deposited (sample b in the Methods section). Figure 4 shows the sample exposed to air and Figure 5 to water. The two figures also show the effect of different directions of linear polarization of the incident light corresponding to the grating orientation. We can see that the image resolution with the grating vector perpendicular to the incident polarization direction is far superior to the resolution with the grating vector parallel to the incident polarization, both in air (where the 10–90% transition for polarization perpendicular and parallel to the grating vector are 3.62 μm and 1.23 μm respectively, see Fig. 4a,c) and water (where the 10–90% transitions for polarization perpendicular and parallel to the grating vector are 5.73 μm and 2.75 μm respectively, see Figure 5a,c). The reason that the images obtained from the perpendicular polarization are superior arises from the fact that the propagation length of the SPs is sufficient for them to sense both coated and uncoated areas^9^. The experimental and simulation results agree well, however, the experimental images obtained show some asymmetry. The difference in edge response on each side of the grating is thought to be caused by surface damage during the lift-off process.

We then matched the experimental results to the simulations from RCWA, using the layer parameters obtained from the calibration experiments with the gold layers exposed to liquid. The first thing to note is that the same sample was used in all four cases (propagation parallel/perpendicular to the grating vector and exposure to air and water respectively). It is crucial that the same BSA thickness can be used to match all the experimental results in order to give confidence that the parameters obtained really correspond to the experimental situation. Comparing the simulated results with the experimental results shown in Figures 4b,d and 5bd, we can determine the thickness of the BSA to be 13.5 ± 0.5 nm both in air and water. The values for the expected measured change in angle on a uniform substrate are shown with the upper and lower horizontal lines in Figs 4b,d and 5,d, we can see that interpreting the experimental results with the simple Fresnel equations even for a grating of this period leads to very large errors especially when the SPs propagate across the grating.

**Figure 6:**
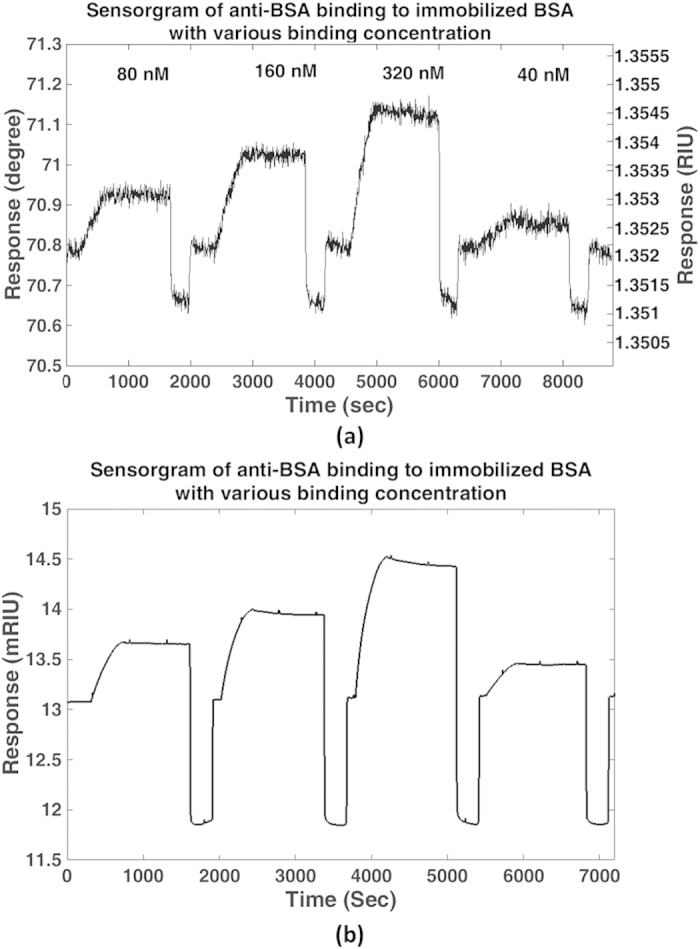
Interaction of BSA with different anti-BSA concentrations with the protein grating produced by photolithography. (**a**) the sensorgram from ASWSPRM (**b**) the sensorgram from SR7000DC.

Figure 6 demonstrates the BSA interactions with different anti-BSA concentrations using the protein grating samples produced by photolithography (samples c as described in the Methods section). We have also used the microcontact printed samples for binding measurements, it was expected that the more limited mechanical interaction with photolithography would produce a more chemically active surface, which justified the more complex sample preparation protocol. Figure 6a shows the ASWSPRM sensorgram of the interaction using SPR angle taken from the mean value obtained from a BSA immobilized region of 625 pixels (4.7 × 4.7 μm^2^). Each image took approximately 3.9 seconds to obtain. We can see that ASWSPRM does not only give us high resolution but the quantitative SPR angle change during the cycles. It can be seen that both ASWSPRM system and the commercial Reichert system (SR7000DC) give similar changes in refractive index. The microscopic images are much noisier but the area of our sensor region is 8 × 10^4^ times smaller than the commercial single point sensor, indeed our SNR compares very well to other widefield SPR microscope systems such as the system described by Vander and Lipson^15^. Their paper gives careful consideration to the variation of sensitivity with the spatial resolution. For instance, they present values of refractive index sensitivity of 10^−2^ RIU for spatial resolution of 1 micron, which was extrapolated to 10^−3^ RIU at low spatial frequencies. Moreover, we expect that further optimization of our microscope system can improve the SNR by at least and order of magnitude, measures such as reference channels to correct for temperature drift and operation at a longer wavelength where the SP dip is much sharper will considerably improve performance.

## Discussion

We have established a new prototype microscope. The key element is a SLM that provides a convenient, fast, precise and stable means of varying or scanning the incident illumination angle. Experimental results show the system has both high spatial resolution and refractive index sensitivity of 3.4 × 10^−5^ RIU. The results also demonstrate that it is possible to use this system to obtain the quantitative changes of the bulk medium refractive index or the thickness of a dielectric film on the gold coated substrate. The system is capable of on-line biological and chemical experiments with far finer spatial resolution than is possible with classical prism based surface plasmon microscopes. We have noted that as expected the lateral resolution is superior perpendicular to the direction of plasmon propagation. Although this effect is not as pronounced at it is with a prism based arrangement it is still an issue. Since one of the merits of the system we have proposed is to be able to study a higher density of analytes it is appropriate to use the available area on the surface as efficiently as possible and for this reason one could use rectangular regions to identify the binding events or, of course, radial polarization^15^.

## Methods

### Measurement principle

We develop a relatively simple and effective approach to obtain high spatial resolution and refractive index sensitivity while performing widefield SP microscopy in aqueous solutions. This allows us to follow binding processes in real time in biologically relevant solutions. In order to achieve this we scan the illumination angle using a SLM to control the illumination distribution in the BFP of the microscope objective. This allows precise control of the incident angle of the illumination path. Moreover, by varying the azimuthal angle we can control the polarization state of the illumination to provide a reference channel, if required. Although the SLM provides a very convenient way of scanning the angle we are essentially faced with another issue that needs to be addressed. The angle at which SPs are excited in water is approximately 71 degrees at 633 nm wavelength which implies that a minimum numerical aperture of 1.44 is necessary. Since we wish to follow the variation of the reflection coefficient around and beyond the resonant angle, it is necessary to access even greater angles. Furthermore, the situation is exacerbated because the refractive index of pure water is at the low end of the refractive indices encountered in biological media. In order to solve this problem it is necessary to use as high NA aperture microscope objective as possible. The highest NA available in a commercially available microscope objective lens is 1.65 (Olympus APO 100x/NA1.65 apochromatic objective lens), however, this requires the use of special high index coverslips fabricated from sapphire with refractive index 1.78. These coverslips are prohibitively expensive for routine use and the immersion fluid, diiodomethane, is moderately toxic and also volatile. There is therefore a considerable motivation to use a microscope objective that can employ standard coverslips (BK7, refractive index 1.52) and conventional immersion oil. For this reason we employed a Nikon (Apo 60 × TIRF, 1.49 Oil). Furthermore, increasing the illumination wavelength from 633 nm to 690 nm reduces the resonant angle by approximately 2 degrees.

### Optical arrangement

**Figure 7:**
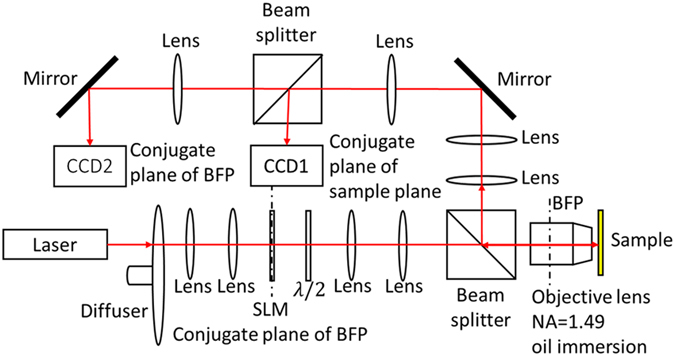
Schematic of the angle-scanning surface plasmon resonance widefield microscope.

Figure 7 is a schematic of the optical system used in our studies. A laser beam (690 nm diode laser) is passed through a rotating diffuser which destroys the spatial coherence of the beam and increases the image quality when averaged over time at the CCD. Images are thus considerably more free of artefacts compared to spatially coherent illumination from a single point in the back focal plane. Single point illumination does allow pure *p-*illumination to be achieved, although, as shown in the results section, the potential improvement in image contrast is not justified by the degradation of image quality. A pair of lenses adjusts the beam size of the laser beam on the SLM which is placed in a plane conjugate with the BFP of the objective lens. The SLM and half wave plate control the intensity and polarization of light separately. CCD1 acquires widefield surface plasmon resonance images and CCD2 images the light distribution in the BFP of the objective. The BFP images help to monitor the alignment of the system, the quality of the sample and the excitation of SPs^18^.

### Incident angle control using the SLM

**Figure 8:**
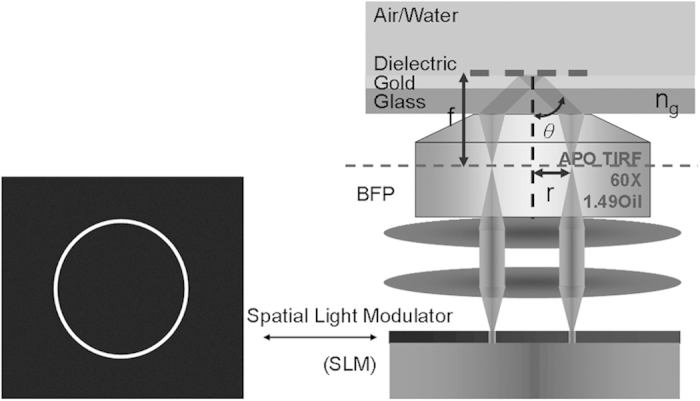
Amplitude Spatial light modulator (SLM) in a plane conjugate to the back focal plane of the microscope objective is used to control the incident angle of the illumination.

Figure 8 shows coverslips coated with a metal layer to support generation of SPs onto which various layers (both continuous and spatially varying) of different refractive index are deposited. The coverslip is usually exposed to air or water. The incident angle, *θ*, of the light at the glass-gold interface which can be calculated through the sine condition of the optical system


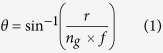


where *r* is the radius of an annulus in the BFP, *n*_*g*_ is the refractive index of the glass substrate and *f* is the focal length of the objective lens.

An 8-bit liquid crystal SLM with SVGA resolution (800 × 600 pixels), a 60Hz frame rate and a pixel pitch of 33 μm was used. In our experiments the full width of the back focal plane was imaged to 500 pixels in the back focal plane. This means that an annulus one pixel wide covers a change in the sine of the incident angle equal to: 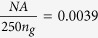. This is approximately 0.3 deg. when the sample is in air with incident angle ≈43 deg. and 0.6 deg. when the sample is immersed in an aqueous medium with incident angle ≈69 deg. This is considerably narrower than the width of the dip, so does not substantially broaden the angular response of the system. The width of a single pixel does not, however, limit the smallest increment of angle that we can impose to the radius of the arc since the center of gravity of the ring can be altered using the available grey levels of the SLM, so a sub-pixel movement may be imposed by changing the transmission of adjacent pixels so the value of one pixel increases while the other decreases, as the total light transmission remains constant.

### Data processing and image reconstruction

**Figure 9:**
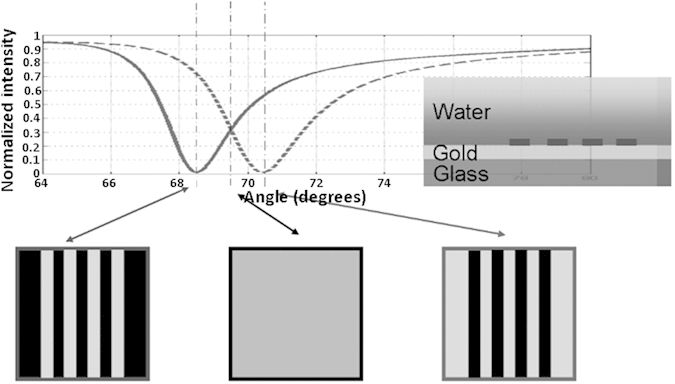
Schematic showing the contrast of coating reflected light image with different incident angles with 690 nm illumination.

Figure 9 shows the basic principle behind the measurement. Consider a uniform layer of 50 nm of gold deposited coated with a 20 nm deep BSA, all immersed in water. The solid line is the reflected intensity for a BK7-gold-water structure. At larger angles of incidence the coated region appears dark, while at smaller incident angles the bare region is dark; the contrast of the grating structure is thus inverted with incident angle. At intermediate incident angles the contrast will disappear. Tracing out the curve at each pixel will allow us to reconstruct the local refractive index, although to interpret the results properly it is necessary to take account of the local environment as the response is not the same as for a uniform sample^9^.

By scanning the illumination angle, a series of widefield surface plasmon images are obtained as a function of incident angle. By plotting intensity against incident angle *θ* for each pixel location, we can estimate the resonant plasmon angle as a function of spatial position. We can calculate the plasmon angle by fitting a 3^rd^ order polynomial at each pixel off-line and calculating the resonant angle from the zero of the differential. Finally, a median filter is used to remove outliers from the image, this replaces the pixel value by the median value of a 5-by-5 neighborhood of the pixel, thus providing smooth angle-scanning widefield surface plasmon resonance images (ASWSPRM).

### Sample preparation

**Figure 10:**
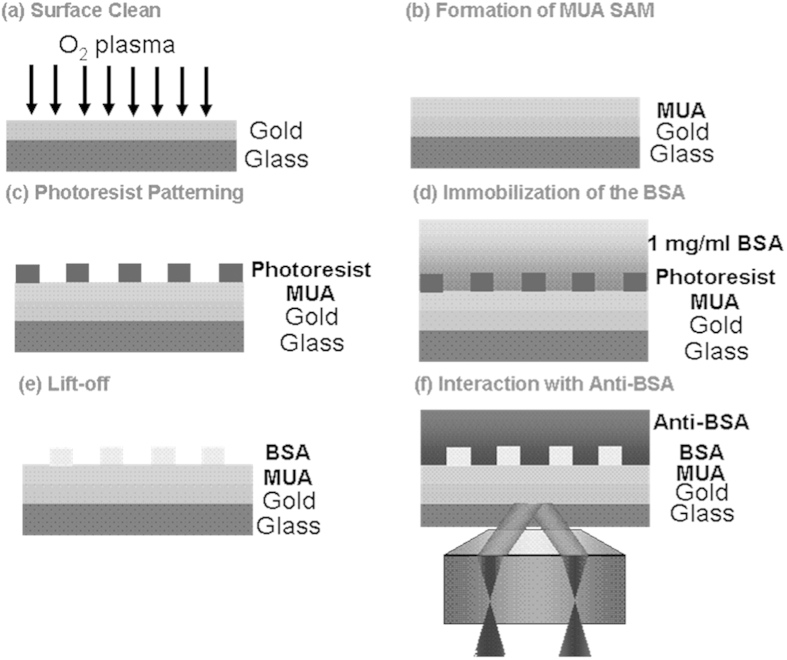
The procedure to produce BSA grating patterns covalently immobilized on gold surface created by photolithography.

All the samples were coverslips (Fisherbrand) coated with a very thin layer (1–2 nm) of chromium over coated with approximately 50 nm of gold by Thin Metal Films Ltd. Three different types of sample are used to form images:

- Bare gold-coated coverslips. The coverslip is placed in a cuvette and exposed to liquids of different refractive index. Starting with distilled water isopropanol (IPA) was added in small increments to increase the refractive index. The concentration of IPA was increased by 0.8% (wt.) every 300 seconds, resulting in a refractive index increase of 6.67 × 10^−4^ RIU.
- BSA grating patterns on gold surface were produced by microcontact printing. A poly(dimethylsiloxane) (PDMS) stamp was made by standard soft lithography techniques^19^. A drop of 3% BSA (Sigma) in distilled water onto the surface of the stamp, the excess solvent was removed in a stream of nitrogen. The BSA was stamped directly on the gold surface by holding it in a fixed position for 30 seconds. After the BSA was dry, the sample could be exposed to either air or water.
- BSA grating patterns were covalently immobilized onto the gold using photolithography surface created by photolithography. This process is essentially similar to the process described in Kausaite *et al.*^20^ to pattern protein on a glass surface, here, of course, we pattern the protein onto the gold. This allowed the protein to be bound to its complementary antibody so that localized interactions could be studied^20^. Figure 10 shows the process. The gold surface was cleaned in an oxygen plasma (60 Watt, 10 min) then immersed in 1 mM 11-mercaptoundecanoic acid (MUA, Aldrich) in ethanol (Sigma) for 16~24 hours to form a MUA self-assembled monolayer (SAM) on gold surface. The sample was then rinsed with ethanol and distilled water and dried with nitrogen. A positive photoresist (BPRS-150, OCG Chemical Ltd) was spun on MUA SAM (3500 rpm, 30 seconds) and soft-baked in an oven for 30 min at 110 °C. The chip was exposed through a grating photomask on a mask aligner (CA-800, Cobilt) with a broadband mercury lamp for 65 seconds and developed (PLSI:water 1:3) for 30 seconds. After rinsing with distilled water, the chip was activated in 0.1 M N-(3-dimethylaminopropyl)-N’-ethylcarbodiimide (EDC, Thermo) and N-hydroxy succinimide (NHS, Fluka) mixed in water for 5 min and rinsed with PBS+500 μl/l Tween 20 (PBST, Sigma). The EDC/NHS activation was repeated three times and the sample was immersed in 1 mg/ml BSA in PBST for 15 min to immobilize the BSA on the chip. After rinsing with PBST and deactivated with 1 M ethanolamine (Sigma) in water for 5 min, the chip was soaked in acetone (Sigma) for 10 seconds to lift off the photoresist. Thereafter, the sample was rinsed with PBST immediately and soaked in 50 mM HCl (Fisher) for 5 min to stabilize the BSA. After the BSA (ligand) was patterned, we put the sample into a homemade flow cell on our microscope and set the syringe pump flow rate to 60 μl/min to perform the interaction cycle with anti-BSA rabbit IgG (analyte, Invitrogen). This process was as follows: (1) Flow the PBST and wait for the system to stabilize. (2) Flow 80 nm anti-BSA in PBST for 7 minutes for the association phase. (3) Flow the PBST at least 16 minutes for the dissociation phase. (4) Flow the 50 mM HCl for 5 minutes to regenerate the interaction complex. The interaction cycle (1)~(4) was repeated with different anti-BSA concentrations(160 nM, 320 nM, 40 nM).

### Computer simulation

In this section we describe rigorous diffraction analysis of the SP microscope. The purpose of the simulations is twofold, firstly, to examine the performance of different configurations of the microscope and, finally, to use the simulations to compare with the actual measurements obtained with the microscope.

Rigorous coupled wave analysis (RCWA) was used to calculate microscope response for the grating sample on gold substrate (10 nm thick of 24 μm grating period with 10 μm of BSA and 14 μm gap width) in order to compare with experimental results. The full aperture of the objective lens was modelled with 501 × 501 array points having its value of *k*_*x*_ and *k*_*y*_ corresponding to its incident angle and azimuthal angle. Reflected electric and magnetic fields of each point on the back focal plane were calculated using in-house RCWA software package written in MATLAB which fully utilizes multi core processors thus facilitating parallel computing. The field results were then stored and post-calculated as described below. For RCWA technique, the accuracy of the results depends the number of diffracted orders included in the calculation; the greater the number of diffracted orders calculated, the more accurate the results, provided numerical instabilities are not introduced by the near singularity of the matrices^9^,^10^. Calculation of greater numbers of diffracted orders, of course, comes at expense of computation time and memory. For the problem described above, the number of diffracted orders included in the calculation was 401 to achieve a smallest feature size of 24 μm/201≈120 nm. In essence, incident waves from each position in the back focal plane were used as incident angles, the diffracted order for each incident beam was calculated from RCWA and the *x* and *y* components of the back scattered diffracted electric field was then placed in the correct position in the back focal plane. Beams from each incident angle retained their coherence, but diffracted light arriving at a similar position in the back focal plane from different incident angles were summed incoherently since the ground glass removes the spatial coherence across the input plane. The effect of scanning the annulus was simply simulated by summing over the range of incident angles appropriate for each angular position. More details explaining the calculation of the microscope response using RCWA are given in refs 9 and 10.

## Additional Information

**How to cite this article**: Tan, H.-M. *et al.* High Resolution Quantitative Angle-Scanning Widefield Surface Plasmon Microscopy. *Sci. Rep.* **6**, 20195; doi: 10.1038/srep20195 (2016).

## References

[b1] Salvi J. & Barchiesi D. Measurement of thicknesses and optical properties of thin films from Surface Plasmon Resonance (SPR). Appl. Phys. A 115, 245–255, 10.1007/s00339-013-8038-z (2014).

[b2] Jamil M. M. A. *et al.* High resolution imaging of bio-molecular binding studies using a widefield surface plasmon microscope. Sensors and Actuators, B: Chemical 129, 566–574, 10.1016/j.snb.2007.09.006 (2008).

[b3] Yeatman E. & Ash E. A. Surface plasmon microscopy. Electronics Letters 23, 1091–1092, 10.1049/el:19870762 (1987).

[b4] Kano H. & Knoll W. Locally excited surface-plasmon-polaritons for thickness measurement of LBK films. Optics Communications 153, 235–239, 10.1016/S0030-4018(98)00240-5 (1998).

[b5] Byrne G. D. *et al.* Total internal reflection microscopy for live imaging of cellular uptake of sub-micron non-fluorescent particles. Journal of Microscopy 231, 168–179, 10.1111/j.1365-2818.2008.02027.x (2008). 18638200

[b6] Zhang B., Pechprasarn S. & Somekh M. G. Quantitative plasmonic measurements using embedded phase stepping confocal interferometry. Optics Express 21, 11523–11535, 10.1364/OE.21.011523 (2013). 23670009

[b7] Zhang B., Pechprasarn S. & Somekh M. G. Surface plasmon microscopic sensing with beam profile modulation. Optics Express 20, 28039–28048 (2012). 23263039 10.1364/OE.20.028039

[b8] Zhang B., Pechprasarn S., Zhang J. & Somekh M. G. Confocal surface plasmon microscopy with pupil function engineering. Optics Express 20, 7388–7397, 10.1364/OE.20.007388 (2012). 22453418

[b9] Pechprasarn S. & Somekh M. G. Surface plasmon microscopy: Resolution, sensitivity and crosstalk. Journal of Microscopy 246, 287–297, 10.1111/j.1365-2818.2012.03617.x (2012). 22497520

[b10] Pechprasarn S. & Somekh M. G. Detection limits of confocal surface plasmon microscopy. Biomedical Optics Express 5, 1744–1756, 10.1364/BOE.5.001744 (2014). PMC4052908 24940537

[b11] Pechprasarn S., Zhang B., Albutt D., Zhang J. & Somekh M. Ultrastable embedded surface plasmon confocal interferometry. Light: Science and Applications 3, e187, 10.1038/lsa.2014.68 (2014).

[b12] Somekh M. G., Stabler G., Liu S., Zhang J. & See C. W. Wide-field high-resolution surface-plasmon interference microscopy. Opt. Lett. 34, 3110–3112, 10.1364/OL.34.003110 (2009). 19838242

[b13] Zhang J., See C. W., Somekh M. G., Pitter M. C. & Liu S. G. Wide-field surface plasmon microscopy with solid immersion excitation. Applied Physics Letters 85, 5451–5453, 10.1063/1.1815391 (2004).

[b14] Huang B., Yu F. & Zare R. N. Surface Plasmon Resonance Imaging Using a High Numerical Aperture Microscope Objective. Analytical Chemistry 79, 2979–2983, 10.1021/ac062284x (2007). 17309232

[b15] Vander R. & Lipson S. G. High-resolution surface-plasmon resonance real-time imaging. Opt. Lett. 34, 37–39, 10.1364/OL.34.000037 (2009). 19109632

[b16] Rakić A. D., Djurišić A. B., Elazar J. M. & Majewski M. L. Optical properties of metallic films for vertical-cavity optoelectronic devices. Appl. Opt. 37, 5271–5283, 10.1364/AO.37.005271 (1998). 18286006

[b17] Ordal M. A., Bell R. J., Alexander R. W., Long L. L. & Querry M. R. Optical properties of Au, Ni, and Pb at submillimeter wavelengths. Appl. Opt. 26, 744–752, 10.1364/AO.26.000744 (1987). 20454208

[b18] Zhang J., Pitter M. C., Liu S., See C. & Somekh M. G. Surface-plasmon microscopy with a two-piece solid immersion lens: bright and dark fields. Appl. Opt. 45, 7977–7986, 10.1117/12.810702 (2006). 17068536

[b19] Love J. C., Estroff L. A., Kriebel J. K., Nuzzo R. G. & Whitesides G. M. Self-Assembled Monolayers of Thiolates on Metals as a Form of Nanotechnology. Chemical Reviews 105, 1103–1170, 10.1021/cr0300789 (2005). 15826011

[b20] Kausaite A. *et al.* Surface plasmon resonance label-free monitoring of antibody antigen interactions in real time. Biochemistry and molecular biology education: a bimonthly publication of the International Union of Biochemistry and Molecular Biology 35, 57–63, 10.1002/bmb.22 (2007). 21591057

